# Draft Genome Sequence of *Burkholderia ambifaria* RZ2MS16, a Plant Growth-Promoting Rhizobacterium Isolated from Guarana, a Tropical Plant

**DOI:** 10.1128/genomeA.00125-16

**Published:** 2016-03-17

**Authors:** Bruna Durante Batista, Lucas Mitsuo Taniguti, Claudia Barros Monteiro-Vitorello, João Lúcio Azevedo, Maria Carolina Quecine

**Affiliations:** Department of Genetics, Luiz de Queiroz College of Agriculture, University of São Paulo, Piracicaba, São Paulo, Brazil

## Abstract

*Burkholderia ambifaria* strain RZ2MS16 was isolated from the rhizosphere of Amazon guarana in Brazil. This bacterium exhibits a remarkable capacity to promote the growth of corn and soybean. Here, we report the draft genome sequence of RZ2MS16 and some genes related to multiple traits involved in plant growth promotion.

## GENOME ANNOUNCEMENT

The genus *Burkholderia* comprises 98 species (http://www.bacterio.net/burkholderia.html), which are distributed in diverse habitats. The ecological versatility of *Burkholderia* spp. is due to their large genomes, often composed of several large replicons (two to four circular chromosomes and large plasmids). An important variation in genome size from 4 to 8 Mbp is observed in the *Burkholderia* genus (1). Several species are components of rhizosphere and have been frequently described due to their potential for biological control (2, 3) and plant growth promotion (4–6). These findings have increased interest in using *Burkholderia* species isolates as agricultural bioinoculants.

The strain presented here, *Burkholderia ambifaria* RZ2MS16, is a rhizobacterium isolated from guarana (*Paullinia cupana*), a typical plant from the Amazon region. This bacterium has been characterized *in vitro* regarding some traits related to plant growth promotion, such as biological nitrogen fixation, indole acetic acid (IAA) production, and siderophore production. *In vivo* assay showed that RZ2MS16 was significantly able to promote corn and soybean growth (B. D. Batista, submitted for publication). Here, we describe the draft genome sequence of strain RZ2MS16 to better understand its genetic background, which may provide important clues to the development of sustainable bioinoculants.

Genomic DNA was isolated using the DNeasy blood and tissue kit (Qiagen, USA) and sequenced at the Center of Functional Genomics (ESALQ/USP, Brazil) using Illumina MiSeq. Approximately 7 million paired-end reads with a mean size of 250 bp (coverage of ~150-fold) were assembled using A5-miseq software (release 20140604) (7), resulting in ~8 Mb of the RZ2MS16 genome. The draft is composed of 82 scaffolds, with an average size of 98,730 bp, *N*_50_ contig size of 242,048 bp, and GC content of 65.73%. Gene prediction was performed by PROKKA (v1.12) (8), resulting in 7,270 open reading frames, with an average size of 949 bp, 736 of which were predicted as secreted proteins.

The genome of RZ2MS16 presents genes related to plant growth promotion, such as siderophores (9), including several genes involved in iron acquisition, transport, metabolism, and storage, and the uptake regulation protein (*Fur*). The annotated genome also revealed 21 genes involved with production of IAA, a plant hormone associated with root elongation (10). A few genes were identified that are related to nitrogen fixation, and the prediction of *vnf* nitrogenase suggests that the bacterium uses the vanadium nitrogenase system.

The annotated genome has 65 genes responsible for motility, including 47 genes for flagellar motility. Thirteen genes were found related to N-acylhomoserine lactone and 9 genes involved with transcriptional regulation of the quorum-sensing system. Of the genes that were assigned, 1-aminocyclopropane-1-carboxylate (ACC) deaminase activity may also potentially contribute to plant growth promotion of strain RZ2MS16 (11).

These data may explain the plant growth promotion ability of strain RZ2MS16. The genome information will facilitate formulations for its practical application as a potential inoculant with a broad host range. Further analyses are in progress and will be presented separately.

### Nucleotide sequence accession numbers.

This whole-genome shotgun project has been deposited at DDBJ/EMBL/GenBank under the accession number LKPJ00000000. The version described in this paper is the first version, LKPJ01000000.
